# A Toolbox for Quantitative Gene Expression in *Varroa destructor*: RNA Degradation in Field Samples and Systematic Analysis of Reference Gene Stability

**DOI:** 10.1371/journal.pone.0155640

**Published:** 2016-05-16

**Authors:** Ewan M. Campbell, Catriona H. McIntosh, Alan S. Bowman

**Affiliations:** Institute of Biological and Environmental Sciences, University of Aberdeen, Aberdeen, United Kingdom; Swedish University of Agricultural Sciences, SWEDEN

## Abstract

*Varroa destructor* is the major pest of *Apis mellifera* and contributes to the global honey bee health crisis threatening food security. Developing new control strategies to combat *Varroa* will require the application of molecular biology, including gene expression studies by quantitative real-time reverse transcription-polymerase chain reaction (qRT-PCR). Both high quality RNA samples and suitable stable internal reference genes are required for accurate gene expression studies. In this study, ten candidate genes (succinate dehydrogenase (SDHA), NADH dehydrogenase (NADH), large ribsosmal subunit, TATA-binding protein, glyceraldehyde-3-phosphate dehydrogenase, 18S rRNA (18S), heat-shock protein 90 (HSP90), cyclophilin, α-tubulin, actin), were evaluated for their suitability as normalization genes using the geNorm, Normfinder, BestKeeper, and comparative ΔCq algorithims. Our study proposes the use of no more than two of the four most stable reference genes (NADH, 18S, SDHA and HSP90) in *Varroa* gene expression studies. These four genes remain stable in phoretic and reproductive stage *Varroa* and are unaffected by Deformed wing virus load. When used for determining changes in vitellogenin gene expression, the signal-to-noise ratio (SNR) for the relatively unstable genes actin and α-tubulin was much lower than for the stable gene combinations (NADH + HSP90 +18S; NADH + HSP90; or NADH). Using both electropherograms and RT-qPCR for short and long amplicons as quality controls, we demonstrate that high quality RNA can be recovered from *Varroa* up to 10 days later stored at ambient temperature if collected into RNAlater and provided the body is pierced. This protocol allows the exchange of *Varroa* samples between international collaborators and field sample collectors without requiring frozen collection or shipping. Our results make important contributions to gene expression studies in *Varroa* by proposing a validated sampling protocol to obtain high quality *Varroa* RNA and the validation of suitable reference genes for expression studies in this globally important pest.

## Introduction

Half of all insect-mediated pollination of crops is provided by managed honey bee colonies, representing a significant economic benefit to global agricultural [1,2]. The ectoparasitic mite, *Varroa destructor* is *the* major contributory factor to declines in honey bee health and subsequent global colony losses. *Varroa* has spread from its host species, the Asian honey bee, *Apis cerana*, in South East Asia, through the international trade in honey bees and now has a worldwide distribution on the European honey bee, *Apis mellifera* [3–5]. *V*. *destructor* feed on developing bee larvae and pupae within sealed brood cells [6]. Feeding causes direct damage to the bee brood and *Varroa* also vector a host of pathogenic viruses, including deformed wing virus (DWV) [7–10]. The global spread of *Varroa*, coupled with the vectoring of pathogenic DWV has resulted in a decline in honey bee health worldwide [3,11]. Many regions now experience higher than average winter losses of honey bee colonies [12,13] and colonies infested with *Varroa* are likely to perish within 3 years in the absence of treatments or intervention [14]. Integrated pest management strategies for mites include chemical application of acaricides as well as practices such as drone brood removal, organic acid treatments and, in the longer term, breeding of tolerant honey bees [6,15,16]. Many mites are now resistant to current acaricides, necessitating the development of new treatments as a matter of urgency [17].

The release of the *Varroa* genome in 2010 [18] along with an increase in transcriptomic resources has enabled researchers to begin to explore physiological pathways involved in the *Varroa* / host / virus axis [19,20]. *Varroa* mites depend on honey bee brood to reproduce and have evolved a complex and intimate strategy that demands precise temporal host-sensing and reproductive cues [21,22]. Molecular technologies such as RNA-interference (RNAi) [23, 24], quantitative PCR [25] and RNAseq gene expression assays (Campbell *et al* unpublished) are increasingly important and viable means of investigating *Varroa* host-sensing, feeding and reproduction and are the primary tools in identifying and developing effective therapeutic targets. Analysis of gene expression data from qPCR studies rely on the quantification of multiple housekeeping, or reference, genes that show a stable transcription level across different samples [26][27]. Normalization of expression data with unstable reference genes can dramatically alter the perceived level of target gene regulation, leading to erroneous interpretation and results [28–30].

The primary aim of this study was to evaluate potential reference genes for use in qPCR studies for *V*. *destructor*. Both reproductive and phoretic stage mites were compared alongside mites with high and low titres of DWV to examine “treatment” effect on reference gene stability. Brood and phoretic mites can be physiologically differentiated and are increasingly being studied with regards to reproductive cues and host sensing by gene expression studies [22]. In addition, it has become more evident that research on DWV infection and transmission must increasingly involve the *Varroa* vector and as such it is vital to understand whether virus load has an effect on reference gene stability [8,19,31].

A primary challenge in this post-genomic era is the preservation and storage of high quality arthropod samples from demanding and remote field sites, and collections by non-laboratory trained personnel or from multiple collaborators across the globe [32]. It is therefore vital that pathogen, pest and entomological research utilises the best sampling and storage techniques available to produce high quality RNA for subsequent analysis.

As there is currently no artificial rearing system to de-couple *Varroa* mites from honey bee colonies, all *Varroa* mites for use in laboratory experiments are collected from managed honey bee colonies in the field prior to applying molecular techniques in the laboratory. It is fundamental that *Varroa* RNA to be used in transcriptomics or gene expression experiments is of sufficiently high quality for downstream gene expression analysis [26]. RNA is extremely prone to degradation due to endogenous RNases found in all organisms and also exogenous RNases in the environment [32–34]. This study explored the stability and quality of *Varroa* RNA collected and stored under a range of conditions to provide a proven sampling protocol for researchers.

Within the post-genomic *Varroa* era it is vital that researchers can both isolate high quality RNA from samples and then quantify target gene transcripts accurately. Results provided by this study will be essential tools for post-genomic research in *Varroa* mites. qPCR, RNAseq and transcriptomic studies in *V*. *destructor* and the continued development of therapeutic and genetic approaches for control will benefit from the outcomes of this research.

## Material and Methods

### Biological samples and cDNA synthesis

Adult female *V*. *destructor* mites were collected from hives at the University of Aberdeen apiary in Newburgh, Aberdeenshire (Grid Ref NK004271). Phoretic mites were collected by ethanol wash from 500 adult bees [35]. Reproductive phase mites were collected by opening and removing individual mites from larval brood cells. RNA was extracted from individual mites using the ZR micro-RNA kit (Cambridge Bioscience, Cambridge, UK), DNAse treated with RQ1 and RNA quantified using a Nanodrop ND-1000 microspectrophotometer (Thermoscientific, Loughborough, UK). 200 ng RNA was reverse-transcribed using iScript cDNA synthesis kit for each sample (Biorad, Hemel Hempstead, UK). Resultant cDNA was quantified using a Nanodrop ND-1000 and the concentration adjusted to 5 ng/μl with RNase-free water.

To assess the stability of the candidate reference genes as influenced by DWV viral load, we studied cohorts of mites that either had very low or very high titres of DWV. These studies were performed only on phoretic mites. All the mites had some DWV, but the level varied greatly. To separate mites into high and low viral samples, individual mite cDNA was assayed for levels of DWV by qPCR, according to the method of Yu *et al* (2005); employing a standard curve of a plasmid containing a 1520bp fragment of DWV for absolute quantification [36]. Depending on the virus titre, mites were separated into High Level (> 12 million gene copy equivalents per mite) or Low level (< 400 gene copy equivalents) DWV. Details of virus quantification methods and DWV titres of varroa included in the study are given in Supporting Information (S1 Method). cDNA from individual mites was pooled into triplicate groups of 2 mites for the treatments high virus and low virus treatments. cDNA from individual mites was pooled into triplicate groups of 8 mites for the phoretic and reproductive treatments.

### Gene selection and primer design

Candidate reference genes were selected on the basis of transcripts previously used as housekeeping genes for qPCR analysis in *Varroa* mites, along with genes commonly used in qPCR in insects and other Acari [37][38][39][40]. In total 10 genes were initially considered from a variety of functional classes (Table 1). The primers for RT-qPCR (Table 1) were taken either from the literature or designed from genomic database transcripts using primer3 plus (http://www.bioinformatics.nl/cgi-bin/primer3plus/primer3plus.cgi/) and modified manually. Melting curve analysis and standard curves showed that these primers were within expected efficiency parameters and had annealing temperatures suitable to a standardised plate run.

**Table 1:** Candidate reference genes selected for stability analysis.

Transcript	Function	Oligo sequence	Product size	Tm^a^(°C)	E^b^ (%)
**Succinate dehydrogenase (SDHA)**	Respiratory chain and citric cycle enzyme	F: AAACCGGGAACGACCTTATC	108bp	58.4	94
		R: TCCAATCCTTCCAACTGTCC			
		LF: AAACCGGGAACGACCTTATC	449bp	58.4	84
		LR: TCTCACGTGCCATCACAATC			
**NADH dehydrogenase (NADH)**	Respiratory chain enzyme	F: TCCGCTTAAGGAGCTTATCG	72bp	58.4	103
		R: ATCACGCACAGCAGGTTATC			
**Large Ribosomal sub-unit (LRSU)**	Structural protein in ribosome	F: ACGTATTCCATTCGGCTTCC	106bp	58.4	102
		R: GAGTCCGGCGAGGTATGAGT			
**TATA-binding protein (TBP)**	DNA transcription factor	F: AAGATCGTCAACGTGCTTGG	74bp	58.4	94
		R: TTGTTGGCCTGTGAGAAAGG			
		LF: TCAACCCGTCTTCTTCCTTG	551bp	61.2	101
		LR:CAACCTCTGAGGCTCACAAAC			
**Glyceraldehyde-3-phosphate dehydrogenase (GAPDH)**	Glycolysis	F:CGCAAGGCCGGTGCCAAAAA	62bp	62.1	99
		R:ACGAACATTGGCGCATCGGGT			
**18S rRNA (18S)**	Structural protein in ribosome	F: AATGCCATCATTACCATCCT	60bp	54.3	97
		R: CAAAAACCAATCGGCAATCT			
**Heat shock protein 90 (HSP90)**	Chaperone protein	F: TTTGTAACCGACACGAGCTG	117bp	58.4	92
		R: TGTTGAGCGTGTGAAGAAGC			
		LF: GATTTCGAGGTGCTTCTTCG	578bp	58.4	81
		LR: ACATGAAGCTGGGAATCCAC			
**Cyclophilin**	Ubiquitous multi function protein	F: CCGGTAAAGCAGTCCACGA	98bp	59.5	103
		R: AGGCATCGACTTTCCTTTGG			
**α-tubulin**	Cytoskeletal structure	F: AATTAGTTGCTCGCCACGAT	241bp	56.4	104
		R: TGGCAAGAGGACTTCCCATA			
**Actin**	Cytoskeletal structure	F: TTGCTGACCGTATGCAGAAA	100bp	54.6	94
		R:CCGATCCAGACGGAATACTT			

^a^ Tm is annealing melting temperature for oligos.

^b^ E is efficiency of qPCR reaction as determined by standard curve slope.

### Standard curve construction and Real-Time PCR

RT-qPCR was performed on a CFX96 Real-Time PCR Detection system using iTaq universal SYBR® Green supermix (Bio-Rad, UK). Reactions were run in 20 μl volumes consisting of 10μl iTaq supermix (BioRad), 4μl water, 5μl (5ng/ul) of template cDNA and 1μl (2 mM) respective primers. qPCR cycling conditions were as follows: 1 cycle of 3 min at 95°C, followed by 40 cycles of 10s at 94°C and 30s at the primer specific annealing temperature (Table 1). Melting curve analysis was 5s incremental increases of 0.5°C from 65°C to 95°C. Control reactions with primer and template free reaction mixtures were included. Three biological and three technical replicates were performed for each sample. A serial dilution of total combined cDNA pools was used to obtain standard curves and the corresponding primer amplification efficiency for each gene calculated. Cq values were extracted from CFX Manager software and analysis of melting curves was performed to confirm correct profiles for each gene transcript reaction. Efficiency was calculated from a standard serial dilution curve utilising CFX manager software.

### Data analysis and ranking of candidate reference genes

Three software programs were used to evaluate reference gene stability; geNorm (version 3.4) [41], Normfinder (version 0.953) [42] and Bestkeeper (version 1.) [43]. Cq values are transformed to relative quantities using the delta-Ct method for geNorm and NormFinder analyses. Cq values were used directly for calculations for BestKeeper analysis. In addition to software programs, the delta Cq value was calculated to assess stability without taking into account efficiency data of primers [44].

geNorm was used to rank the reference genes by calculating the gene expression stability value M by assessing the mean pairwise expression ratio for each reference gene against all other tested reference genes [45][41]. Relative quantities, using the highest expression value for calibration, were imported to geNorm software. M values were then calculated using the assumption that the expression ratio between two reference genes is identical across samples despite experimental changes or treatments. After each iteration of the algorithm, genes with the highest valued M values are removed and M values are re-calculated for remaining reference genes until only two remain. It is recommended that an M value threshold of 1.5 is used to identify reference genes with a stable expression across samples tested [41]. In addition to M value stability rankings, geNorm software can determine the minimum number of reference genes necessary to accurately normalize qPCR data by generating a normalization factor (NF). NF is determined by taking the geometric mean of the expression levels from the most stable reference genes and then additively recalculating with each next most stable reference gene. The pairwise variation, Vn/Vn+1, between two sequential normalization factors is then calculated to determine the effect of each newly added gene to the NF. Additional reference genes are included in the normalization factor until additional genes give no significant effect. The optimum number of genes is the lowest number of genes with Vn/Vn+1 < 0.15.

NormFinder software log transformed data utilising Cq values and efficiencies taken from qPCR for its primary input data. NormFinder measures reference gene stability by initially determining intra- and inter-group variations between user specified samples, groups and experimental treatments [42]. It then uses these measures of variation to give an overall measure of variation in expression for each individual reference gene. The reference gene candidates with the lowest stability values are the most stable.

BestKeeper was used, alongside geNorm and NormFinder to determine the most stably expressed reference genes. Raw Cq values are imported into Bestkeeper software and a geometric mean of reference gene Cq values is then used to create a Bestkeeper run index [43]. Individual reference genes are then compared to this index based on the coefficient of correlation to the BestKeeper Index. In addition, BestKeeper calculates standard deviation and the coefficient of variation of all candidate reference genes based on Cq values [43].

Analysis was initially carried out globally to indicate reference genes that were stable, and therefore could be utilised, across treatment groups (i.e. phoretic vs. reproductive and Low virus vs. High virus). In addition, analysis was undertaken within treatment groups to determine if a specific reference gene or set of genes is more suitable in studies of a sub-class of mite.

### Assessment of sampling and storage conditions on RNA quality

To examine the effects of RNA degradation on transcripts, RNA quality was assessed after sampling and storing mites under field-realistic conditions. Mites were collected from brood cells [35] and either left intact (unpierced) or their bodies pierced with a fine gauge needle (Gauge#26) before being submerged in 200 μl RNAlater (Life Technologies, Paisley, UK). The tubes were then placed at 4°C for 12 h to allow the solution to thoroughly penetrate the tissues, as per the manufacturer’s recommendation. Samples were removed from 4°C and stored at room temperature for 0, 3 or 10 days to mimic typical times in transit for mailing nationally or internationally. Finally, samples were placed at -80°C until processed. Additionally, some mites that had dropped from honey bee colonies onto sampling trays below the hive were collected after 0, 24h and 48 h [46]. These mites were pierced and placed in 200 μl RNAlater, as above, and stored at -80°C.

RNA was extracted from pooled mite samples (8 mites per extraction) by crushing mites using a micro-pestle in 100 μl RNA lysis buffer and processed using a ZR micro-RNA kit (Zymo Research, Germany). RNA was eluted in 10 μl RNAse-free water. Total RNA concentration was measured using an ND-1000 Nanodrop micro-spectrophotometer (Thermo-scientific, USA). Degradation of the RNA samples was assessed electrophoretically on an Agilent 2100 Bioanalyzer. RIN values are not valid for many arthropod species, including *Varroa*, due to an 18S/28S break in ribosomal RNA [34]. An electropherogram was created for each sample to visually assess for RNA degradation.

Good quality, nascent RNA consists of long RNA strands whereas poor quality, degraded RNA contains a higher proportion of shorter fragments [47]. By comparing the ratio of abundance of short: long products for the same gene after qPCR it is possible to assess the level of degradation and, hence, the RNA quality. All samples (200 ng RNA) were reverse transcribed, as described above. For the genes HSP90, TBP and SDHA, primers were designed for both long (>400 bp) and short (<120 bp) portions of sequence (Table 1). qPCR was performed on equal quantities of cDNA. Reactions were run in 20ul volumes as described above. qPCR cycling conditions were as follows: 1 cycle of 3 min at 95°C, followed by 40 cycles of 10s at 94°C and 30s at the primer specific annealing temperature (Table 1). Melting curve analysis was 5s incremental increases of 0.5°C from 65°C to 95°C. Control reactions with primer and template free reaction mixture were included. Three biological and four technical replicates were performed for each sample. Efficiency and Cq values were calculated using CFX manager software, as described above. The ratios of Cq values for the short: long products for samples stored at room temperature for 3 and 10 days were compared to those at 0 days by Student’s *t*-test.

## Results

### Candidate reference gene amplification efficiency and expression profile

**Fig 1:**
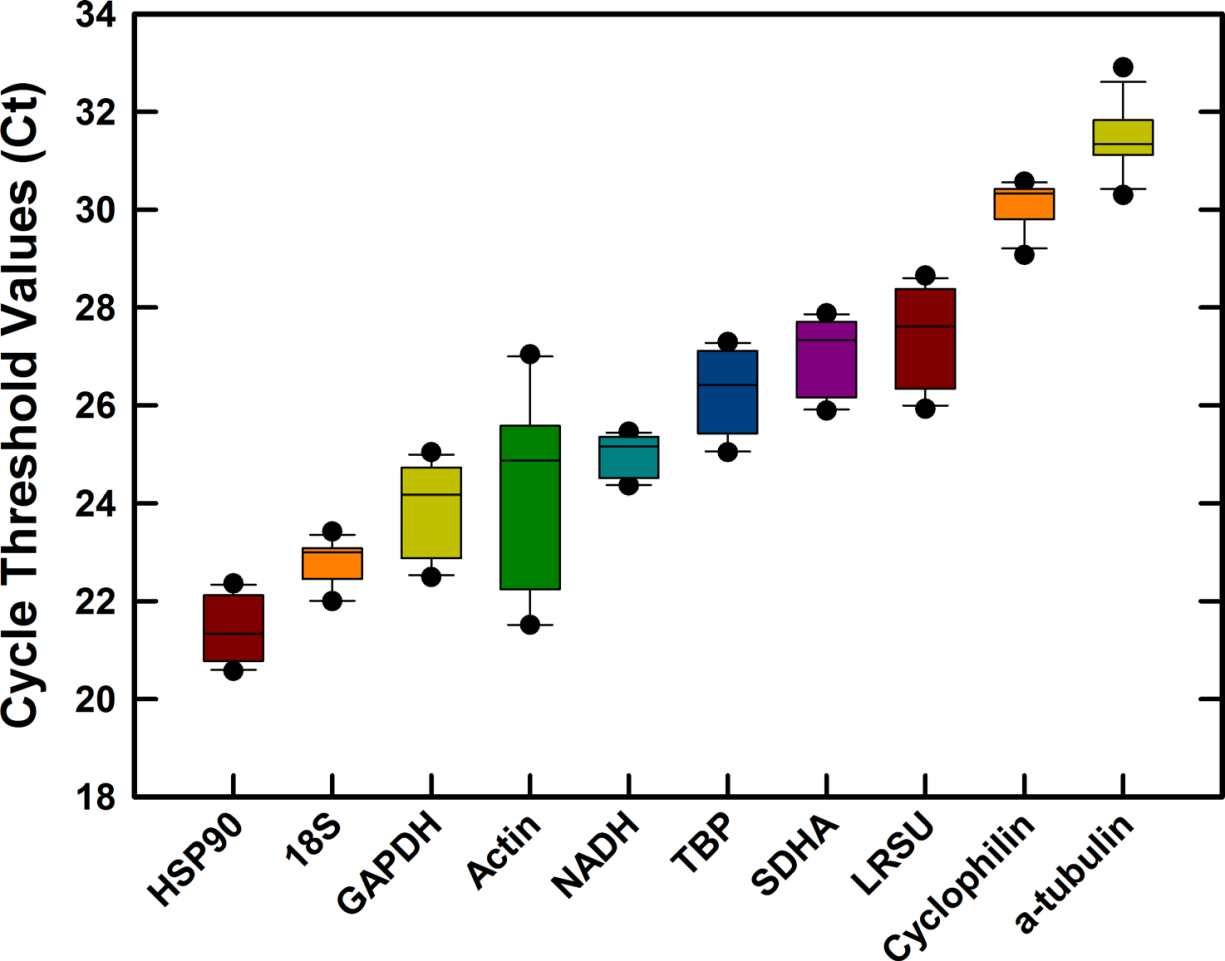
Stabilities of cycle threshold (Cq) values of ten candidate reference genes in *Varroa destructor*. The box plot with medians indicates the 25th and 75^th^ percentiles and the whiskers (error bars) indicate the 10^th^ and 90^th^ percentiles. Black dots represent outliers. Data for each gene from 3 replicates of each grouping (brood, phoretic, Low Virus and High Virus), each sample contained 8 varroa.

Primers were designed for 10 candidate reference genes commonly used as reference genes in acari and other arthropods. Amplification efficiencies of primer pairs ranged from 94–104% (Table 1) indicating efficient qRT-PCR systems for *Varroa*. The specificity of each primer pair was confirmed by melting point analysis showing single products and a single PCR amplicon of the correct size (Table 1) on agarose gels (data not shown). Cq values across all treatment samples (Mean ± SD, n = 12) for the 10 reference gene candidates varied from 21.40 ± 0.64 (HSP90) to 31.43 ± 0.66 (α-tubulin) (Fig 1). The most abundant gene transcripts were HSP90, 18S and GAPDH (Cq <25) whereas cyclophilin and α-tubulin were least abundant (Cq>30) out of those tested. Excluding actin, the variation about the mean was extremely tight across all of the candidate reference genes (coefficient of variation < 3.8%). Actin expression demonstrated a degree of variation (CV = 7.9%), between 2x and 5x that of the other candidate genes suggesting that actin may not be suitable as a reference gene. This result was confirmed in the subsequent systematic analysis by reference gene software below.

### Expression stability assessment analysis by geNorm

**Fig 2:**
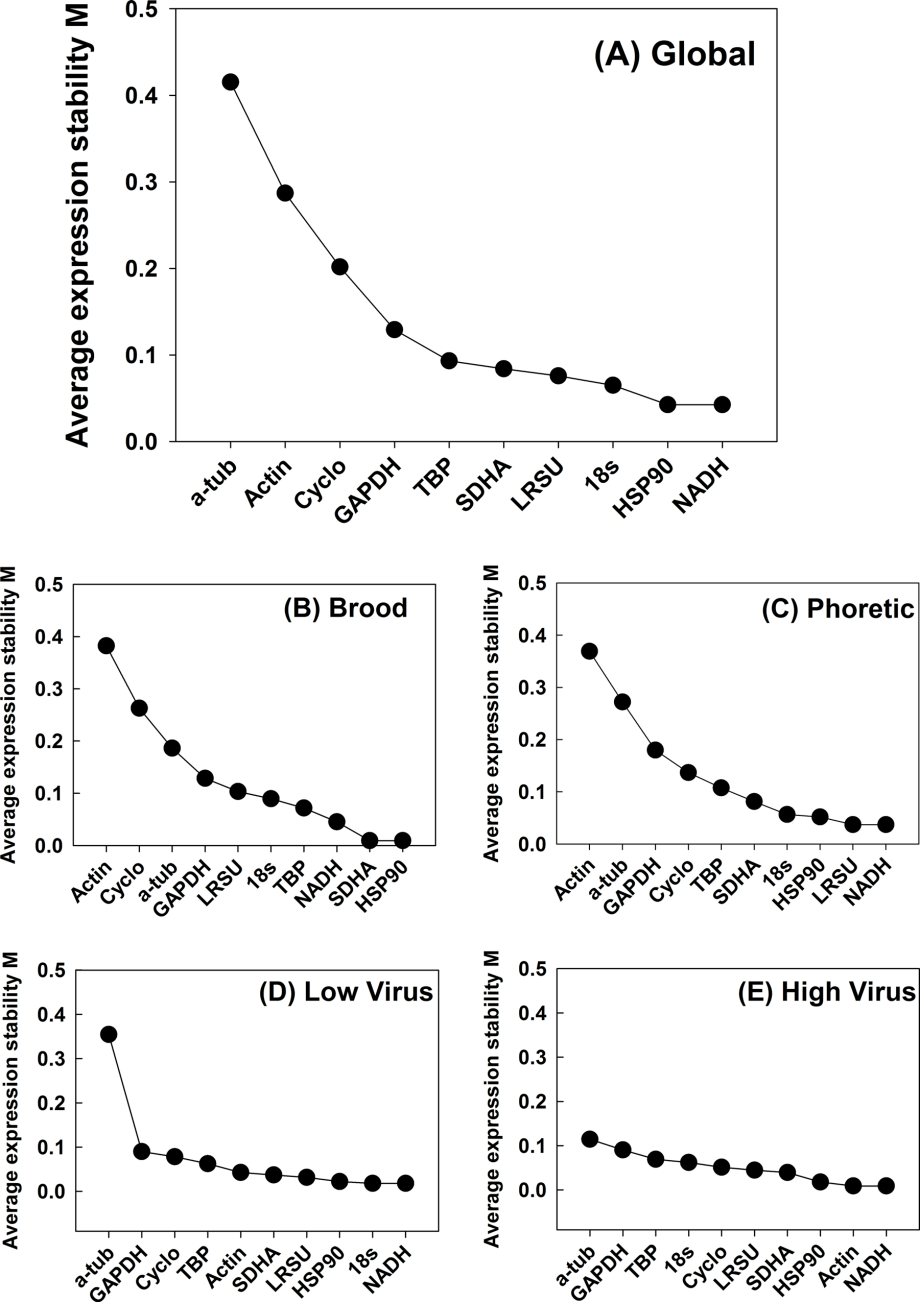
Average expression stability and ranking of ten reference genes. Values are average expression stability (M) as calculated by geNorm(3.4) on a global analysis of all samples (A) as well as for subset analysis on brood mite samples (B), phoretic mites (C), low titre virus mites (D) and high titre virus mites (E).

geNorm is an Excel-based program that evaluates gene expression stability based on log2 transformed expression ratios for pair-wise gene combinations [41,48]. geNorm gives two useful outputs, the first measures reference gene stability based on an indicator of stable expression across samples (M value) and the second measures the optimal number of candidate genes needed for normalization in a gene expression assay. Genes exhibiting an M value ≤ 1.5 are acceptable as reference genes [41]. Initially, the algorithm outputs gene combination standard deviation (V value) followed by a step-wise reduction of genes exhibiting the highest V value and recalculating V until only two genes remain. The average V for a given gene is an indicator of stability expression value M. All 10 candidate *Varroa* reference genes tested in this study were well below the threshold value of M = 1.5 and, thus, deemed acceptable. The lowest M value across all *Varroa* samples (global) was 0.274 for HSP90, indicating the highest stability. Actin and α-tubulin exhibited the highest M values tested at 0.632 and 0.929, respectively, but are both still below the threshold of M ≤ 1.5. When considering *Varroa* across all the groups (i.e. global), in step-wise reduction, the two genes exhibiting the lowest variation and highest stability were HSP90 and NADH with an M value of 0.04 (Fig 2). The least stable genes were actin and α-tubulin (Table 2). When considering individual sample groups (i.e. brood, phoretic, high and low virus groups), both NADH and HSP90 consistently ranked among the three most stable genes tested with M values < 0.1.

**Table 2:** Ranking of candidate reference genes.

Ranking	geNorm	M	NormFinder	SV	Bestkeeper	SD	ΔCq	SV	Overall Ranking
**1**	HSP90	0.274	NADH	0.096	18S	1.322	NADH	0.66	NADH
**2**	18S	0.279	18S	0.182	NADH	1.328	SDHA	0.69	18S
**3**	NADH	0.283	Cyclo	0.209	Cyclo	1.355	TBP	0.71	SDHA
**4**	SDHA	0.284	SDHA	0.229	α-tubulin	1.453	HSP90	0.71	HSP90
**5**	LRSU	0.286	HSP90	0.231	HSP90	1.478	18S	0.73	TBP
**6**	TBP	0.291	TBP	0.299	SDHA	1.655	Cyclo	0.75	Cyclo
**7**	GAPDH	0.394	GAPDH	0.427	TBP	1.721	GAPDH	0.79	LRSU
**8**	Cyclo	0.500	LRSU	0.492	GAPDH	1.794	LRSU	0.81	GAPDH
**9**	Actin	0.632	α-tubulin	0.541	LRSU	1.935	α-tubulin	1.08	Actin
**10**	α-tubulin	0.929	Actin	1.132	Actin	3.128	Actin	2.47	α-tubulin

M, global average expression stability (geNorm); SV, stability value (Normfinder); SD, standard deviation of Cq values (Bestkeeper); SD, standard deviations (comparative delta-Ct method). The Overall Ranking gives a global ranking using all four methodologies in order of greatest stability from 1 to 10.

### Expression stability assessment analysis by NormFinder

NormFinder determines inter- and intra- group variation of expression values for each gene [42]. The expression values for this analysis were calculated from Cq values using the Pfaffl method which accounts for the amplification efficiency [49]. NormFinder algorithm is a model-based approach for estimation of expression variation that takes into account intra- and inter-group variations for normalization factor calculation. NormFinder produces a stability value for each gene which represents variation in expression across samples and between groups. Calculated stability values ranged from 0.096 (NADH) to 1.132 (actin) across all sample groups (Table 2). The best combination of two genes suggested is 18S and cyclophilin with a stability value of 0.109. Within the DWV treatment groups the most stable gene was SDHA (0.056) whereas within the phoretic and reproductive mites NADH (1.11) was most stable (data not shown). Across all treatments, actin is the only candidate gene tested that would be unsuitable for qPCR as it was above the stability value threshold of 1.0.

### Expression stability assessment analysis by Bestkeeper

Bestkeeper identifies the most stable reference genes [43] using raw Cq values and amplification efficiencies to determine the most stable reference genes. Unstable genes are removed from analysis and the remaining genes are then used to generate the Bestkeeper index. A pairwise correlation analysis is performed between each combination of genes in the Bestkeeper index. From this analysis, the reference genes can be ranked from most stable to least stable using the standard deviation output (Table 2). Global analysis of samples places 18S (SD = 1.32) and NADH (SD = 1.33) as the leading reference genes. When an analysis of individual sample groups (i.e. brood/phoretic, high/low virus groups) was carried out, NADH was consistently ranked in the top four reference genes at SD = 1.28 and SD = 1.08, respectively. 18S was the most stable gene in the DWV assay samples (SD = 1.05) but interestingly was ranked as one of the least stable genes in brood/phoretic samples (SD = 1.46) alongside actin and GAPDH.

### Expression stability assessment analysis by ΔCq

In addition to the above programs, a standard ΔCq analysis was carried out [44]. This method does not take into account the different efficiencies of reaction primer sets but gives a useful raw measure of stability and yielded similar results to the more complex algorithms with NADH ranked as the most stable reference gene analysed (Table 2).

### Overall identification of optimal reference genes

Data from ΔCt was combined with ranking data for geNorm, NormFinder and Bestkeeper to compile an overall ranking list of stability (Table 2) showing that the best candidate reference genes recommended for global gene expression studies in *Varroa* are NADH, 18S, SDHA and HSP90.

### Optimal number of reference genes

**Fig 3:**
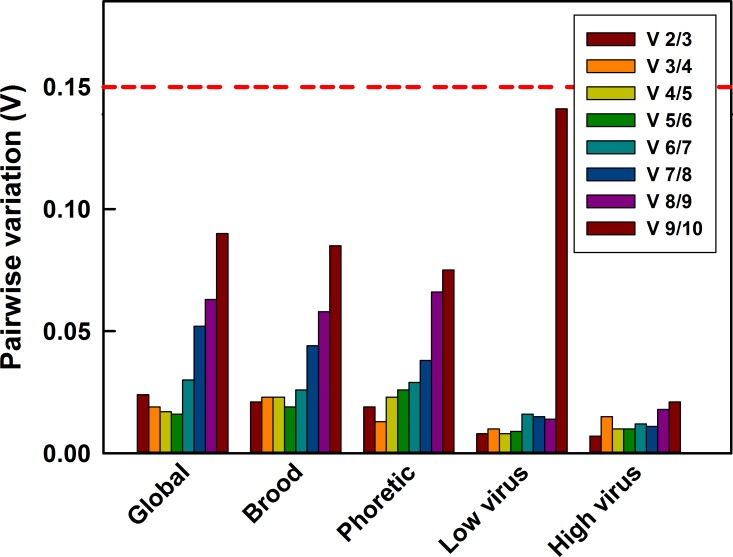
Pairwise variation analysis to determine optimum number of reference genes for accurate normalization. A pairwise variation analysis (geNorm) globally for all samples (n = 12) as well as subset analysis on brood mite samples (n = 3), phoretic mites (n = 3), Low virus mites (n = 3) and High virus mites (n = 3). Each sample comprised 8 pooled mites. Pairwise variation is compared with 0.15, below which the inclusion of a subsequent reference gene is not necessary to ensure accurate normalization.

The number of candidate genes needed for accurate normalization in gene expression experiments was analysed in geNorm by a pair-wise variation analysis between the normalization factors Vn/Vn+1 [41]. In a global analysis of mites from all the sample groups and for individual sample groups, V2/3 is lower than the cut-off value of 0.15 in all the scenarios (Fig 3). Hence, no third control gene would be required when performing RT-qPCR in *Varroa* in any of the sample groups, suggesting that using the two most stable genes (HSP90 and NADH) are sufficient for normalization of qPCR data. The lowest pairwise variation across the global analysis was V5/6 suggesting five reference genes would be optimal, but given the extremely low values at V2/3 then using any more than two genes would result in minimal gain. When pair-wise variation analysis was done considering individual sample groups (i.e. brood, phoretic, high and low DWV groups), V2/3 values were also lower than the threshold value of 0.15.

### Sampling and storage effects of RNA integrity

**Fig 4:**
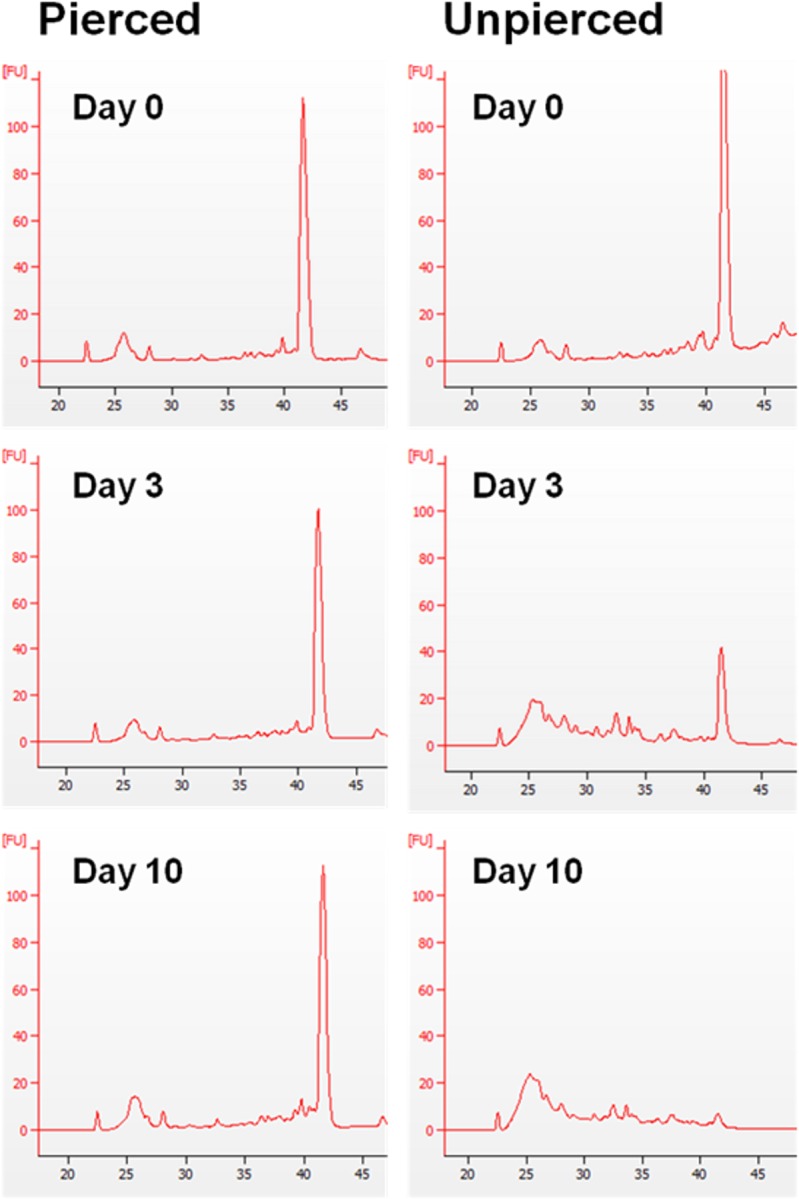
Electropherogram assessment of RNA integrity from pierced and unpierced *Varroa* mites collected into RNAlater after different storage conditions. Total RNA was extracted from either intact or pierced mites stored in RNAlater for 0, 3 and 10 days at room temperature. 50ng aliquots of RNA were run on the Agilent Bioanalyzer 2100 microfluidics gel analysis platform to generate electropherograms. Single 28S ribosomal peaks are seen in intact samples. Smaller peaks at 22, 26 and 28s are Agilent normalization markers and gel size ladders.

The correct preservation and storage conditions of samples can have major impacts on the quality of extracted RNA and therefore subsequent RNA-based applications such as qPCR, RNAseq and RNAi interpretation [50,51]. To investigate the impact on RNA quality of collecting mites from field samples, such as from “mite trays” [35], or from mites collected and stored in RNAlater under different storage regimes, we performed RNA extraction and analysis using a silica bead and column based kit that yields highly concentrated RNA from small insects and arthropods. The Agilent 2100 Bioanalyzer is a microfluidic capillary electrophoresis systems and is the current gold standard for RNA quality assessment [52]. The Agilent 2100 analyzes 18S and 28S ribosomal peaks in RNA samples, displaying clear and distinct bands for both these large rRNA peaks in high quality samples and giving a RNA Integrity Number (RIN). Many arthropods, however, show a single rRNA peak instead of the two clear peaks expected for the two large rRNA species, 18S and 28S [34]. The electropherograms for *Varroa* in all samples tested were concurrent with this anomaly (Fig 4). The electropherograms of RNA extracted from mites pierced and stored in RNAlater for up to 10 days all showed a similar RNA profile with no appreciable degradation visible. In contrast, mites that were not pierced showed an appreciable accumulation of low size fragmented RNA by day 3 and almost all larger RNA transcripts were visibly fragmented by day 10 suggesting low RNA quality (Fig 4). Mites that had died within the hive, fallen to the hive floor and then collected 24 or 48 hr later before being placed in RNAlater with pierced bodies had badly degraded RNA (data not shown).

Although visual inspection of electropherograms from pierced RNAlater stored samples showed no variation in degradation (Fig 4) we carried out qPCR on three abundant reference genes to investigate if longer transcripts were being degraded over time. qPCR was performed on samples using long product or short product oligos for HSP90, TBP and SDHA (Table 3). There was no significant change in Cq-value ratios between long and short products over time in any of the three gene transcripts assayed, indicating that degradation of RNA is not an issue for downstream applications.

**Table 3:** Assessment of RNA integrity by the relative abundance of short and long qPCR products of three stable reference genes over differing storage times in pierced *Varroa* in RNA-Later at room temperature.

Gene	Time (Days)	Short Product (Ct value)	Long Product (Ct value)	Short: Long Ratio	*P* ^a^
	0	16.75 ± 0.37	22.97 ± 0.90	0.73 ± 0.007	-
**HSP90**	3	17.04 ± 0.13	23.30 ± 0.53	0.73 ± 0.008	>0.05
	10	17.68 ± 0.40	24.45 ± 0.71	0.72 ± 0.003	>0.05
	0	23.39 ± 0.01	25.32 ± 0.42	0.92 ± 0.005	-
**TBP**	3	23.56 ± 0.91	25.99 ± 0.23	0.91 ± 0.007	>0.05
	10	23.30 ± 0.91	25.72 ± 0.26	0.91 ± 0.005	>0.05
	0	22.71 ± 0.55	22.72 ± 0.47	1.00 ± 0.002	-
**SDHA**	3	23.34 ± 0.53	23.18 ± 0.36	1.01 ± 0.004	>0.05
	10	23.52 ± 0.24	23.32 ± 0.11	1.01 ± 0.003	>0.05

Data presented as Means ± SD, n = 3.

^a^ Short: Long ratios compared against values on Day 0 by Student’s *t*-test

### Validation of reference genes in a case study of vitellogenin expression

**Fig 5:**
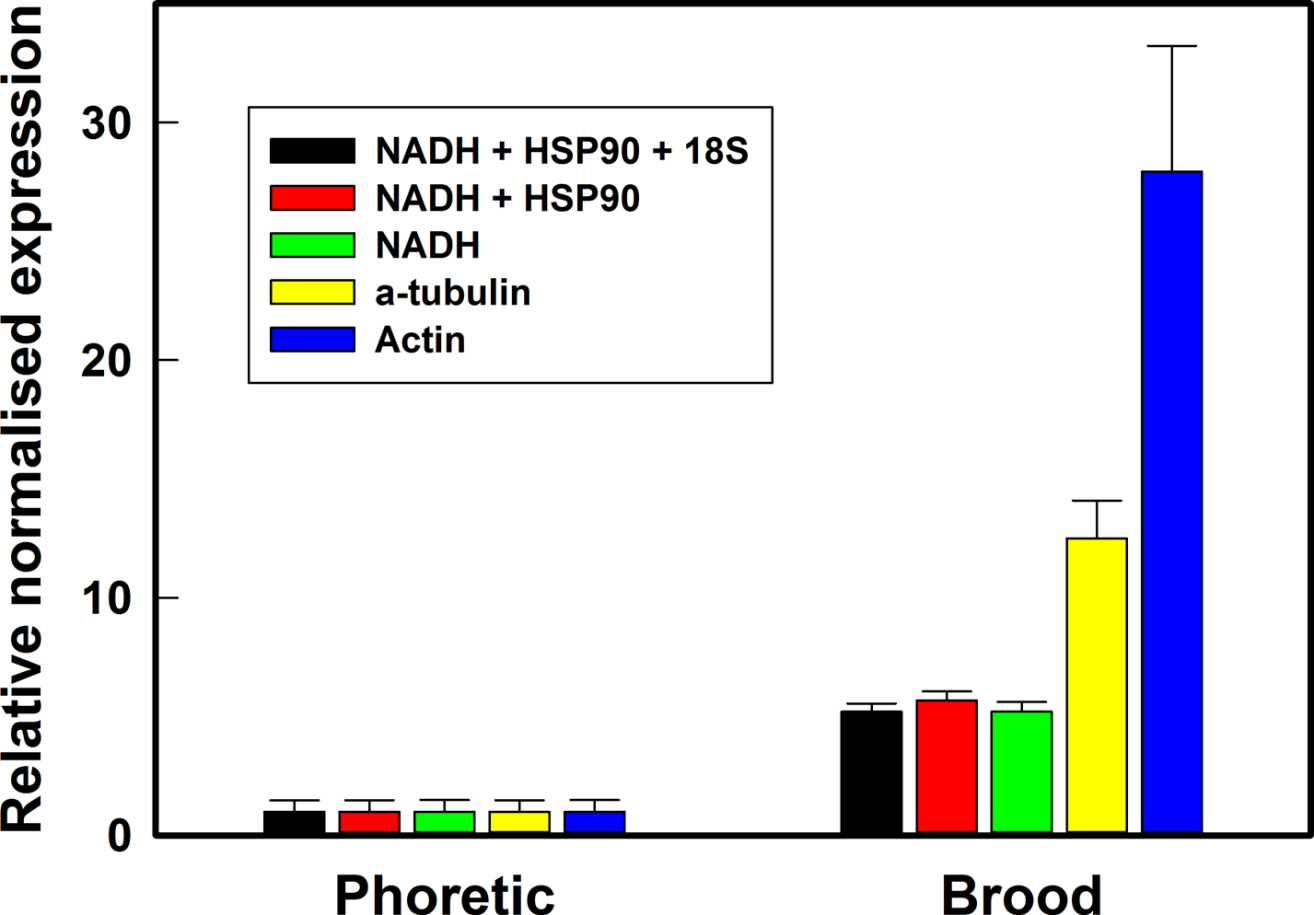
Validation of reference gene selection in a study of vitellogenin gene expression. Expression levels vitellogenin transcript was measured in brood phase mites (reproductive) relative to levels in phoretic mites (non-reproductive) using a combination of the most stable three (NADH + HSP90 + 18S), two (NADH + HSP90) and single (NADH) reference genes, and the least stable reference genes (actin and α-tubulin). Data are Means ± SEM, n = 3.

To examine how the best and worst reference genes from this current study perform under experimental conditions, we carried out gene expression analysis using qPCR on a target gene, vitellogenin, that has been shown to be upregulated significantly in reproductive stage, brood, mites [22]. We found that expression levels of vitellogenin mRNA were significantly increased in brood phase mites using all combinations of candidate reference genes tested (Fig 5). The expression level of vitellogenin normalized against either the single best reference gene, NADH, or a combination of two (NADH and HSP90) or three (NADH, HSP90 and 18S) best reference genes was fairly constant at 5.21-, 5.69- and 5.82-fold, respectively significantly different (P < 0.05) from the least stable reference genes, α-tubulin and actin (12.52- and 27.93-fold, respectively). The coefficient of variation for the normalized fold change in vitellogenin using the best reference gene combinations ranged 11.4–13.6% whereas it was much higher with the unstable genes α-tubulin (21.9%) and actin (32.8%).

## Discussion

The stability of reference genes, especially under variable experimental conditions, can have an enormous impact on the interpretation of qPCR data and it is predicted that qPCR and gene expression profiling will become a critical tool in the field of *Varroa* biology. Previous qPCR studies on *Varroa* have relied on actin [20,22,25], 18S and GAPDH [53] as normalizing reference genes. Reference gene stability has, until now, not been systematically reviewed in the *Varroa* mite although has been investigated in three other pest mite species; *Panonychus citri* [54], *Tetranychus cinnabarinus* [55] and *Tetranychus urticae* [56]. Here we have performed a systematic analysis of housekeeping genes for gene expression studies in *Varroa*. The RNA expression stability of potential reference genes for the ectoparasitic mite, *Varroa* was examined using targets commonly employed in mite, tick and arthropod qPCR studies [37,40,54–57]. The expression stability of 18S, LRSU, actin, a-tubulin, GAPDH, NADH, SDHA, cyclophilin, TBP and HSP90 were assessed in brood, phoretic, high and low virus titre mites.

The expression stability of the 10 candidate reference genes was evaluated using four programs (the ΔCq approach, geNorm, Bestkeeper, and NormFinder). Each program uses different algorithms to evaluate reference gene stability and there were subtle differences in ranking generated by each in the current study. With this in mind a combination of the four model outputs was used to generate an overall ranking, with NADH, 18S, HSP90 and SDHA ranked as most stable. These four most stable genes were consistently the most suitable as reference genes for qPCR in *Varroa* regardless of phoretic or brood stage or DWV load, though the effect of other viruses on these reference genes would need to be ascertained. Notably, 18S was also amongst the most stable genes in *T*. *urticae* [56]and *P*. *citri* [54]. These results suggest that the stability of some housekeeping genes is common across multiple mite species, a result that may inform future qPCR expression studies in other mite and acari pests. Our study proposes to use no more than two of the four references genes NADH, 18S, HSP90 and SDHA as normalizing genes in *Varroa* gene expression studies.

Actin, α-tubulin and GAPDH are the least stable candidates and should not be used as normalizing genes in *Varroa* studies. Actin has been used in a number of studies as a reference gene, including in *Varroa* [22,25]. Actin is the most common protein in eukaryotic cells [58] and in some studies it has been shown to vary considerably in abundance, dependent on developmental stage, age and growth of cells and tissues [59]. Actin was shown to be one of the least stable reference genes in three other mite species; *P*. *citri* [54], *T cinnabarinus* [55] and *T*. *urticae* [56]. In the present study, we showed that actin transcript abundance in *Varroa* was unstable across a range of treatments. Hence, we would not recommend actin as a reference gene in expression studies in *Varroa*.

The use of appropriate and stable reference genes can greatly impact on the accuracy of results in gene expression studies. We used an example of a highly regulated reproductive gene, vitellogenin, to investigate the impact of using stable and unstable reference genes on the interpretation of gene expression results in *Varroa*. Adult female *Varroa* undergo vitellogenesis during egg production when they produce vitellogenin proteins that are laid down as yolk, a major source of nutrients during embryo development [60]. Vitellogenin transcript is found at a low level in phoretic mites but increases in the reproductive phase or “brood” phase [21,22,25]. Results in the present study confirmed vitellogenin upregulation in brood mites and this result was seen regardless of the reference genes used. When the most stable genes were used in combination or singly (NADH + HSP90 +18S; NADH + HSP90; or NADH alone), we obtained very similar results in the increase of vitellogenin (5.2; 5.7 and 5.2-fold, respectively). When the least stable genes were used (α-tubulin; actin) this varied greatly (12.8- and 27.9-fold, respectively). Importantly, the signal-to-noise ratio (SNR) for the relatively unstable genes α-tubulin and actin (4.5 and 3.0) was much lower than for the stable genes NADH + HSP90 +18S; NADH + HSP90; or NADH (8.6; 8.7 and 7.3). This demonstrates that using an inappropriate reference gene could lead to a deceptive result, most notably failure to detect changes in expression levels when the SNR is lowered due to the variation or instability of the reference gene. In a temporally expressed gene with large increases in transcript levels, such as vitellogenin, the direction of regulation is unlikely to be misinterpreted by using inherently unstable reference genes but in a target gene where expression is regulated in a more modest manner then poor selection of reference genes could impact significantly on the interpretation of results [61].

The primary considerations when designing qPCR experiments are the quality of the starting material and how to normalize the data [26,62]. Successful, reproducible and biologically robust assessment of qPCR assays depends on these two crucial factors. The MIQE guidelines for qPCR consider quality assessment of RNA as a prominent factor in success [26,27,45,63]. The quality of starting template is arguably the prime factor in determining the reproducibility and interpreting the biological significance of results [47,63–68]. Indeed, it has been shown that large differences can be expected in relative expression levels measured by qPCR due to starting RNA quality alone [66].

**Fig 6:**
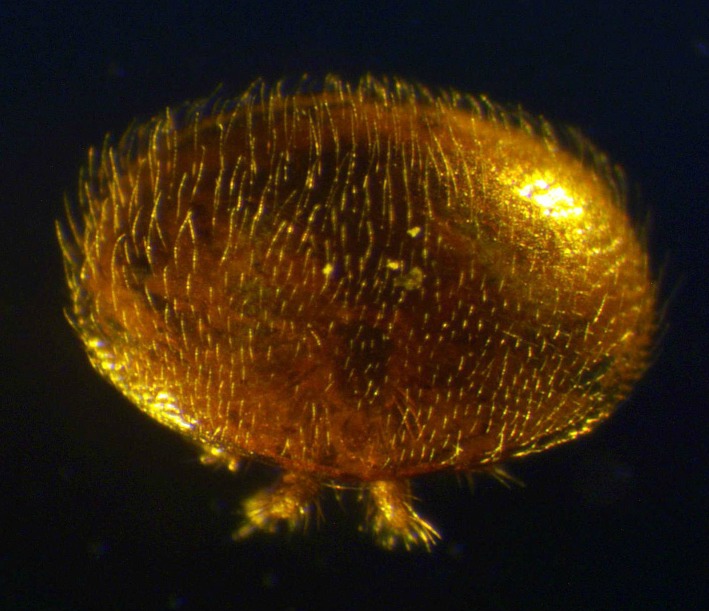
Adult female *Varroa destructor* showing the covering of fine hairs that may prevent RNAlater entering the body.

RNA is inherently unstable and is sensitive to degradation during storage and shipping [32,69,70]. This is particularly important in samples collected in the field, such as *Varroa*, or with other field collected acari and insects, that cannot be cryo-preserved immediately upon collection. RNAlater is a high density salt solution that prevents the degradation of RNA in samples [33,71], but needs to enter the tissues to be effective. Mites and other arthropods are often covered in fine hairs (Fig 6) and this can prevent the RNAlater reaching internal tissues, necessitating the penetration of sample tissues for effective RNAlater entry. In the present study, we demonstrated that it is absolutely vital for researchers to consider degradation during sampling and that *Varroa* collected must be pierced prior to submersion in RNAlater to ensure preservation of RNA integrity. *Varroa* that were not pierced prior to storage in RNAlater had significant RNA degradation after only 3 days at room temperature and would be unsuitable for downstream gene expression studies. However, when sampling is performed as described in this study, notably including piercing of the body, *Varroa* can be stored at room temperature for up to 10 days without any perceivable RNA degradation, as determined by electropherograms and qPCR for short and long amplicons [72]. Ten days represents the time, with some leeway, required to mail samples either nationally or internationally without the need for dry or wet ice between collaborating laboratories or from field sites with limited access to ultracold freezers. Our results indicate that researchers in the fields of *Varroa* biology and physiology can be confident in RNA-based downstream results from samples collected in the field and distributed internationally, if the collection protocols suggested here are followed. Our protocol is not only relevant to *Varroa* but may have implications for the collection of other mites, ticks and small arthropods where piercing of the body is required when storing in RNAlater.

## Supporting Information

S1 Method Details of DWV titre determination in individual mites and allocation to Low and High Virus Group. (DOCX)
