# Supplementary material for: A Toolbox for Quantitative Gene Expression in Varroa destructor: RNA Degradation in Field Samples and Systematic Analysis of Reference Gene Stability
Source: PLoS One. 2016 May 16;11(5):e0155640. doi: 10.1371/journal.pone.0155640 (PMC4868281; doi:10.1371/journal.pone.0155640)
Supplement: S1 Method — (DOCX) [file pone.0155640.s001.docx]

1. **External standard for absolute DWV quantification**

External standards were produced by inserting a fragment of DWV into a plasmid according to methods previously described. An initial 1520bp fragment of the DWV genome was cloned and used to generate a standard curve template for actual qPCR assay (Table 2) (Evans, Schwarz et al. 2013, Genersch 2005, Gisder, Aumeier et al. 2009). A TOPO^®^ TA Cloning kit (Life Technologies, Paisley, UK) was used according to the manufacturer’s instructions to insert the 1520bp DWV fragment into plasmid pCR^®^4-TOPO. Plasmids were cloned into JM109 *E. coli* competent cells, as per the manufacturer’s instruction (Promega, Southampton, UK). Bacterial colonies were grown overnight. Picks were taken from 10 colonies and PCR was done to confirm the presence of the plasmid insert. Positive colonies were grown overnight in LB broth with Ampicillin and the plasmids isolated using a QIAprep^®^ Spin Miniprep Kit (Qiagen, Manchester, UK).

**Supplementary table 1**: Product size, primer sequences and reference for both primer pairs used in the production of external plasmid standards with a DWV insert.

| Primer Name | Sequence | Product size | Source |
| --- | --- | --- | --- |
| DWV_Fstd_F | GGACCATCCTTCCAGTCTACGAT | 1520 | (Evans, Schwarz et al. 2013) |
| DWV_Fstd_R | CTGTAGGTTGTGCTCCTGATGAAGA |  |  |
| DWV_ F1/B1_F | CCTGCTAATCAACAAGGACCTGG | 355 | (Genersch 2005) |
| DWV_F1/B1_R | CAGAACCAATGTCTAACGCTAACCC |  |  |

The exact numbers of whole genome equivalents (wge) in the pooled plasmid sample were determined, using calculated mass and concentration of the plasmid to produce standard stock solutions of known quantities. Aliquots of these stock solutions were stored at -20°C until needed to generate standard curves, an example of which is shown in S1 Fig..

**S1 Fig 1.** Standard curve of DWV whole genome equivalents utilising purified plasmids containing DWV fragments of known mass and concentration.

1. **Absolute DWV quantification protocol**

SYBR Green fluorescent detection was used and the following qPCR cycling: 15 minutes at 95^o^C, followed by 35 cycles of 15 seconds 94^o^C, 30s seconds of 55^o^C and 15 seconds of 72^o^C, followed by 10 minutes at 72^o^C, and a melt curve from 65^o^C to 95^o^C increases 0.5^o^C every 5 seconds. qPCR using the DWV F1/B1 primers (Table S1) was performed on the stock standards and unknown mite cDNA in triplicate. The assay was linear across the dynamic range tested (R^2^ = 0.993) and could detect as little as 30 genome copies or virus particles.

1. **Allocation of phoretic mites to either High Virus Group or Low Virus Group**

The DWV levels in individual mites were determined by the absolute qPCR method described above. Based on these results mites, exhibiting either very high or very low DWV titres were allocated to the High Virus Group or Low Virus Group (S1 Table) such as there were 3 groups of 2 mites for both groups. Mites exhibiting DWV levels that were not at the extremes of the range were not included in the subsequent studies on the effect of DWV titre on the stability of candidate reference genes.

**Table S1.** Mite samples were allocated into High and Low virus groups according to DWV virus whole genome equivalents (wge) determined by plasmid-DWV standard curve.

| **Mite sample** | **Cq value** | **DWV wge** | **group allocated** |
| --- | --- | --- | --- |
| **1** | **9.11** | **108900000.00** | **DWV high** |
| **2** | **9.28** | **106100000.00** | **DWV high** |
| **3** | **9.38** | **102900000.00** | **DWV high** |
| **4** | **9.9** | **77330000.00** | **DWV high** |
| **5** | **12.66** | **12870000.00** | **DWV high** |
| 6 | 22.29 | 34740.00 | **DWV high** |
| 7 | 24.79 | 7501.00 | not utilised |
| 8 | 25.96 | 3495.00 | not utilised |
| 9 | 27.07 | 2004.00 | not utilised |
| 10 | 27.39 | 1752.00 | not utilised |
| 11 | 27.92 | 1274.00 | not utilised |
| 12 | 28.04 | 987.60 | not utilised |
| 13 | 28.46 | 833.40 | not utilised |
| 14 | 29.1 | 513.60 | not utilised |
| 15 | 29.28 | 545.50 | **DWV low** |
| **16** | **29.53** | **392.80** | **DWV low** |
| **17** | **30.44** | **275.00** | **DWV low** |
| **18** | **30.91** | **173.30** | **DWV low** |
| **19** | **31.37** | **149.00** | **DWV low** |
| **20** | **32.27** | **89.61** | **DWV low** |

**References:**

EVANS, J.D., SCHWARZ, R.S., CHEN, Y.P., BUDGE, G., CORNMAN, R.S., DE LA RUA, P., DE MIRANDA, J.R., FORET, S., FOSTER, L., GAUTHIER, L., GENERSCH, E., GISDER, S., JAROSCH, A., KUCHARSKI, R., LOPEZ, D., LUN, C.M., MORITZ, R.F.A., MALESZKA, R., MUÑOZ, I. and PINTO, M.A., 2013. Standard methods for molecular research in *Apis mellifera*. In V Dietemann; J D Ellis; P Neumann (Eds) The COLOSS BEEBOOK, Volume I: standard methods for Apis mellifera research. Journal of Apicultural Research 52(4): <http://dx.doi.org/10.3896/IBRA.1.52.4.11>

GENERSCH, E., 2005. Development of a rapid and sensitive RT-PCR method for the detection of deformed wing virus, a pathogen of the honeybee (*Apis mellifera*). *Veterinary Journal,* **169**(1), pp. 121-123.

GISDER, S., AUMEIER, P. and GENERSCH, E., 2009. Deformed wing virus: Replication and viral load in mites (*Varroa destructor*). *Journal of General Virology,* **90**(2), pp. 463-467
